# Intravenous Immunoglobulin in the Management of Lupus Nephritis

**DOI:** 10.1155/2012/589359

**Published:** 2012-09-27

**Authors:** Scott E. Wenderfer, Trisha Thacker

**Affiliations:** ^1^Department of Pediatrics, Renal Section, Baylor College of Medicine, 1102 Bates Street, Suite 260, Houston, TX 77030, USA; ^2^University of Houston, Houston, TX 77004, USA

## Abstract

The occurrence of nephritis in patients with systemic lupus erythematosus is associated with increased morbidity and mortality. The pathogenesis of lupus nephritis is complex, involving innate and adaptive cellular and humoral immune responses. Autoantibodies in particular have been shown to be critical in the initiation and progression of renal injury, via interactions with both Fc-receptors and complement. One approach in the management of patients with lupus nephritis has been the use of intravenous immunoglobulin. This therapy has shown benefit in the setting of many forms of autoantibody-mediated injury; however, the mechanisms of efficacy are not fully understood. In this paper, the data supporting the use of immunoglobulin therapy in lupus nephritis will be evaluated. In addition, the potential mechanisms of action will be discussed with respect to the known involvement of complement and Fc-receptors in the kidney parenchyma. Results are provocative and warrant additional clinical trials.

## 1. Introduction

Intravenous immunoglobulin (IVIg) is a biological agent composed of polyclonal antibodies, derived from the plasma of a large pool of healthy donors [1–5]. It has been primarily used to treat hypogammaglobulinemia but has also shown promise in treating autoimmune diseases, inflammatory diseases, and cancer. It is FDA approved for the treatment of idiopathic thrombocytopenic purpura (ITP) and Kawasaki's vasculitis. Several anecdotal reports and a few studies have shown promising results on the effectiveness of IVIg in the treatment of systemic lupus erythematosus (SLE). Its use has been widespread; however, its efficacy has not been clearly established.

The precise mechanisms by which IVIg functions as an anti-inflammatory agent remains debatable (Table 1) [1, 6–8]. The presence of Ig in the preparations with specificity for variable regions of pathogenic autoantibodies (anti-idiotype responses) can allow for direct binding and neutralization of pathogenic effector functions. The effector functions of autoantibodies are mediated by receptors for constant regions of IgG (FcR) or receptors for complement components that bind to antigen antibody immune complexes. Complement receptors and FcR are expressed on lymphocytes, granulocytes, monocytes, and some parenchymal cells and can induce tissue injury once bound to immune complexes containing pathogenic antibodies. Monomeric IgG in IVIg preparations has been shown to antagonize pathologic immune complexes binding to activating FcR [9]. Alternatively, nonspecific polyclonal Ig can form immune complexes that bind to inhibitory-type Fc-receptors [6]. These inhibitory Fc-receptors then dampen the effector functions of the activating-type FcR and complement receptors [10–12]. IVIg can similarly augment the clearance of pathogenic autoantibodies via the reticuloendothelial system (RES). The RES uses complement receptors and Fc-R on circulating erythrocytes and monocytes to target immune complexes to the spleen and liver, where they are eliminated [13–15]. In addition, the expression of inhibitory FcR can be upregulated by IVIg, tipping the balance of activating and inhibitory responses. This can modulate cellular immune responses in addition to humoral responses [16]. Exogenous Ig can saturate binding sites on the neonatal FcR, a related receptor with widespread expression that usually accounts for the long half-life of albumin and Ig molecules in serum, via protection from lysosomal degradation [17]. This can lead to increased degradation of auto-Abs and reduction of auto-Ab titers. Finally, antibodies with a particular specificity that naturally exist in normal individuals may exert anti-inflammatory effects, such as has been described for the neutralization of basophil and B-cell cytokines BAFF and APRIL by IVIg [18]. It is likely that IVIg works in part through each of these proposed mechanisms.

IVIg preparations can vary in composition depending on the source and method of preparation [19]. This may account for variability in effect when used clinically. The purity of IVIg can vary from 90% to 98%, and the elimination of IgA, IgE, and IgM can vary widely. The range of IgA content extends from 2.9 mcg/mL to 200 mcg/mL. The glycosylation of Ig molecules can vary as well, which has recently been shown to be important for IVIg's effectiveness in immunosuppression [1, 20]. Preparations can also vary according to the preservative used. Brands containing maltose and sucrose are associated with an increased risk of renal toxicity. Therefore, several brands are now available that utilize preservatives such as glycine or L-proline instead of sugars. Moreover, the osmolality can range from 240 to 636 mOsm/kg. All commercial preparations contain some detectable titer of autoantibodies, including antiphospholipid and anti-DNA Abs. They also contain anti-idiotype Abs specific for antiphospholipids, anti-DNA Abs. Overall, the several preparations of IVIg available allow for more individualized therapy depending on the patient's needs. 

In order to better understand the risks and benefits of using IVIg for the management of patients with lupus nephritis, a review of the literature was undertaken to abstract the available evidence on efficacy and safety. In addition, the potential mechanisms of action of IVIg that might occur when administered to patients with lupus nephritis are considered by reviewing the literature on parenchymal Fc-receptors in the kidney.

## 2. Methods

Methods used were modeled from other systematic reviews of treatments for lupus nephritis [21–23]. Briefly, studies of any design in English were sought concerning IVIg in the treatment of patients with complications of lupus nephritis or SLE. Free text searches were undertaken to identify eligible reports from MEDLINE/PubMed (to April 2012) using the terms IVIg or “intravenous immunoglobulin” and the terms “lupus nephritis” or “lupus erythematosus.” Additional trials were sought in review articles [2–5] and reference lists of retrieved articles. For completeness, we included randomized trials, cohort studies, and case reports. When it could be determined that the same patients were included in multiple publications, we used the largest body of data, the most informative, or the most recent (as appropriate). Reviews with clinical information published in a fuller form elsewhere were excluded, as were studies in which IVIg was used for treating other conditions.

Two reviewers extracted information from the identified publications independently, and disagreement was resolved by consensus. Information extracted included the number of patients treated, dosing regimens, duration of therapy, additional treatments provided, any demographic information (age, gender, and ethnicity), definitions or classifications of lupus nephritis (WHO was used), study design, efficacy and/or safety outcomes, and drop-out rates. Any definition of lupus nephritis, as provided by authors, was accepted. If studies included patients who did not have lupus nephritis, efficacy information was only used for analysis if reported separately for nephritis patients. Efficacy outcomes sought were those of complete or partial renal response rates as defined by the original authors (mainly urine protein excretion, serum creatinine or creatinine clearance, renal survival, or a combination) and subsequent relapse rates. Adverse events sought included mortality, infections, cytopenias, gastrointestinal problems, amenorrhea, azoospermia, and hospitalization rates. Information of adverse events in all patients receiving IVIg was included as there is no data suggesting that adverse events would be different in patients with or without nephritis [21]. 

## 3. Results

An initial literature search identified 630 potential articles for review, of which 32 were found to address the use of IVIg in lupus nephritis (Table 2). Several treatment regimens using different dosages have been reported, most commonly 400 mg/kg/day for 4-5 daily doses (high dose, standard dosing for ITP), although 400 mg/kg/dose 1 × monthly, or 85 mg/kg/day for 4-5 days (low dose), has also been described. In SLE patients, there is no published data on how long exogenous Ig remains present after administration, and there is a lack of consensus on dosing intervals and the duration of therapy. Most publications fail to specify the source of IVIg administered, which precludes analysis of individual preparation methods. As has been pointed out before, comparison of outcomes between studies is hampered by counting day 1 as first day of treatment, or the day of kidney biopsy, rather than first day of symptoms [24, 25].

The short-term effects of IVIg were reported initially in 1989 in three patients with mild SLE who had been treated with 300–500 mg/kg/dose, 1 dose every 4 weeks [26]. Although this low dose had been effective in some autoimmune neurological diseases, the immediate effects on auto-Ab titers in these SLE patients were modest. When the high-dose regimen (400 mg/kg/day for 5 days) was used, greater reductions in auto-Ab titers were noted [26]. In a cohort study from Germany, 12 patients with mild-to-moderate disease given 2 courses of high-dose IVIG had a decline in anti-dsDNA Abs within 1 week. Antinuclear antibodies and complement protein levels were not affected. Within 6 weeks, improvements were noted in clinical disease activity scores which lasted 5 to 12 months [27]. In an Israeli cohort study, clinical disease scores were also improved in 62 patients receiving low-dose IVIg (~500 mg/kg/dose once every 5 ± 2 weeks for a mean of 6 doses) [28]. Unsatisfactory responses were noted for thrombocytopenia, alopecia, vasculitis, and proteinuria. Based on these reports, dosage appears to be important.

Several case reports were identified describing the use of IVIg for various nonrenal manifestations of SLE. The results described were encouraging (Table 3) but have infrequently led to larger prospective trials [29–37, 39]. Benefits in treating cutaneous lupus have not been reproducible [40]. Cohorts (*n* = 26) with autoimmune hemolytic anemia [41] and with thrombocytopenia (*n* = 59) [42] have been published and showed short-term benefits without sustained responses. Positive effects on achievement of live births have been reported in pregnant women with SLE and antiphospholipid antibodies after treatment with IVIg [38], but randomized controlled trials demonstrated equivalency or even inferiority of IVIg compared to heparin and aspirin [43–45]. However, investigators continue to search for subsets of SLE patients who will benefit from IVIg, and clinicians continue to prescribe it for SLE patients who fail initial therapy regimens. In a cohort study from Israel, 20 patients with SLE and organ specific disease involvement were treated with 1–8 courses of high-dose IVIg [46]. Improvements in clinical disease scores, hypocomplementemia, and autoantibody titers were seen in 80% of patients. However, when looking at organ specific response rates, the improvements were seen more in CNS disease, arthritis, fever, and thrombocytopenia than in proteinuria.

Few studies have followed their patients for long term. A 2012 study from Israel followed patients for a mean of 30 months after initiating therapy [47]. Eleven patients with SLE were treated with high-dose IVIG monthly for 6 months, followed by additional courses given every 2-3 months. At latest followup, 6 patients had complete remission, 3 had partial remissions, and 2 patients were nonresponders, defined by improvements in clinical disease scoring. In responders, IVIG had a significant steroid-sparing effect. Adverse effects were reported in 18% of patients during their first course of IVIg, and 50% of all patients treated. Common adverse effects included headache, fatigue, nausea, visual disturbances, and limb pain. Adverse effects resulted in truncation of 8 courses, and two patients suffered severe effects (seizure, pulmonary embolus).

### 3.1. Salvage Therapy of Refractory Lupus Nephritis

Most studies identified that reported efficacy of IVIg in lupus nephritis restricted entry to patients who had failed their initial induction therapy of IV corticosteroids and cytotoxic agents. Twelve case reports were identified (Table 4). The first cases were published in 1982 in Japan, with subsequent cases from Europe and North America [37, 48–55]. Patients received 1 or 2 courses of high-dose IVIg in combination with corticosteroids, with or without plasma exchange or cytotoxic agents. By biopsy, the responders had class II, III, IV, or V nephritis. Patients recovered renal function with reductions in proteinuria and reduced immune deposits on repeat biopsy. 

Two cohort studies of high-dose IVIg were identified. In an Israeli cohort, 7 patients with biopsy proven class IV or V nephritis were treated with 1 to 6 courses of high-dose IVIg after failing therapy with IV cyclophosphamide and prednisone [56]. All patients had nephrotic syndrome. All 7 experienced decreases in proteinuria and improvement or resolution of nephrotic syndrome. One patient had a complete remission which persisted at least three years. Only one patient had a relapse, which occurred 4 months after discontinuation of the IVIg [56]. In an Italian cohort study, 12 treatment refractory patients with SLE were treated with 6–24 monthly courses of high-dose IVIg [57]. A progressive clinical improvement was observed in 11 patients, associated with increases complement protein levels and decreases in auto-Abs, and marked improvements in renal function and proteinuria.

One cohort study was identified that used a longer course of lower-dose IVIg. In a Bulgarian cohort study of patients with all forms of treatment refractory chronic glomerulonephritis, better outcomes were reported in the 58 patients who had SLE [58]. All patients were treated with a low-dose IVIg regimen, 85 mg/kg/day on alternate days, for a total of three days, repeated quarterly for up to 7 years. At the conclusion of the study, 30% of the patients with lupus nephritis achieved full remission (unchanged or improved renal function, resolution of nephrotic syndrome, and proteinuria <0.5 gram/day) and 40% patients achieved partial remission (unchanged or improved renal function, improvement in nephrotic syndrome, and proteinuria <1.5 gram/day). Of nonresponders, nearly all died or survived with end-stage renal disease (ESRD), indicating the severity of disease in this cohort. Reported adverse effects were fever, chills, nausea, vomiting, headache, and rash, and none occurred in more than 10% of individuals.

Additional cohort studies were identified which involved important subsets of patients with SLE. One study demonstrated efficacy of a single course of IVIg in three patients with acute kidney injury from combined inflammatory nephritis and thrombotic microangiopathy [59]. The only study of IVIg treatment in patients with ESRD involved 2 patients with symptomatic SLE on dialysis treated with high-dose IVIg [60]. Both patients demonstrated clinical and serologic improvement and tolerated the IVIg administration well. There were only transient declines in serum albumin concentrations noted, which might reflect saturation of neonatal FcRs that protect albumin from lysosomal degradation [75]. In the only report of IVIg use in children with lupus nephritis, 9 children with biopsy-proven class IV or V nephritis were treated with high-dose IVIg [61]. These children had not responded to pulse methylprednisolone or intravenous cyclophosphamide. Five of 8 with class IV nephritis saw marked improvement in renal function and decreases in IgG deposits on repeat biopsy, while the remaining three experienced a reduction in their class of nephritis. The sole patient with class V disease had a partial renal response. Occasional fever, chills, hypotension, and rash were reported in these cohorts, but overall prevalence of adverse effects is unclear from the literature.

Overall, the response rates to the various IVIg regimens are promising. However, all of the studies were uncontrolled trials that leave open the possibility that these patients would have done just as well without the IVIg, as there is known to be delayed benefits of IV solumedrol, IV cyclophosphamide, azathioprine, and other immunosuppressants. 

### 3.2. Use of IVIg as Part of Induction Therapy for Lupus Nephritis

Only a few studies were identified that treated lupus nephritis with IVIg as part of the initial therapy. The numbers of patients treated are small, and the doses administered tended to be lower. One case report reported efficacy and safety in a patient with a complement deficiency [62]. As part of the 1999 cohort study from Israel mentioned above, 5 of the 20 patients treated with 1–8 courses of high-dose IVIg had renal involvement, ranging from mild proteinuria or nephrotic syndrome [46]. Improvements in renal disease were noted, but were inferior to those reported in the CNS disease, arthritis, and thrombocytopenia. Patients with nephritis in the 2008 cohort study also showed some response: resolution of urinary casts in 88% of patients, but of proteinuria in only 20% [28]. Conversely, An Italian cohort of three patients with renal flare failed to respond to 2 courses of high-dose IVIg and steroids [63].

In a randomized study of 14 patients with class IV lupus nephritis, IVIg was compared to intravenous cyclophosphamide [64]. Five patients received low-dose IVIg (400 mg/kg) monthly for eighteen months. Nine patients received cyclophosphamide 1 g/m^2^ IV every two months for six months, then every three months for twelve months. The method of randomization was not described, and it is unclear if there was any dropout after randomization. Patients could also be treated with prednisone at the physicians' discretion. After the 18-month treatment period, a marked improvement in renal function (both serum creatinine and creatinine clearance) was noted in both groups. IVIg was not shown to be inferior to this dose of IV cyclophosphamide. However, IVIg had a modest steroid sparing effect over cyclophosphamide.

The utility of IVIg as first line therapy for lupus nephritis, therefore, remains unclear. No study has compared IVIg to the most commonly used IV cyclophosphamide regimens (NIH, Eurolupus protocols) or to mycophenolate. In addition, no study of IVIg as add on therapy with any of these induction regimens has been published. 

### 3.3. The Use of IVIg as Maintenance Therapy for Lupus Nephritis

No studies were identified using IVIg as a maintenance therapy for lupus nephritis. 

### 3.4. Adverse Effects of IVIg in SLE

Most patients with SLE and nephritis tolerated their IVIg therapies. However, deterioration of renal function following IVIG treatment has been recognized [65, 66, 70, 72–74, 76]. A CDC report cited 120 cases of nephrotoxicity worldwide, with only 26% of cases occurring in patients with preexisting renal disease [72]. Incidence has been reported in 10–33% of some cohorts [65, 73]. Determining the etiology of acute kidney injury in patients with SLE can be challenging. The majority of nephrotoxicity cases prior to 2000 (90%) have been attributed to IVIg preparations utilizing the stabilizers maltose and sucrose. Intracellular accumulation of these sugars leads to cellular swelling and vacuole formation in tubular epithelial cells of the kidney [74]. Additional risk factors for renal toxicity following IVIG therapy include age >70 years, renal impairment pre-treatment, and diabetes mellitus [73]. In addition to the risk of AKI, IVIg was associated with renal flares [74]. The risk appears to be greater in children, as 3 of 6 patients treated with IVIg in one pediatric cohort from Toronto developed renal flare [71]. Patients with anti-phospholipids or other thrombophilias should receive aspirin therapy to minimize risks for thrombosis associated with infusions of IVIg [67, 74]. Mild adverse reactions of IVIg are common and include infusion reactions, headache, dermatitis, hepatitis, pseudo-hyponatremia, neutropenia, and aseptic meningitis [47, 68, 69, 74]. Infusion reactions typically respond to slowing down the infusion rate, and the other reactions respond to withdrawal of IVIg infusions. Headaches are less frequent when high dose IVIg is given 400 mg/kg/day over 5 days than when given 1 gm/kg/day over 2 days [46].

Finally, the choice of IVIg preparation can be influenced by the SLE patient's comorbidities. In SLE patients with impaired glucose tolerance or diabetes mellitus, preparations with sucrose or maltose should be avoided to minimize risk of hyperglycemia. Patients with nephrotic syndrome or edema should receive more concentrated Ig preparations, whereas patients with renal insufficiency should receive preparations with lower osmolalities. In SLE patients with effective IgA deficiency, the use of IVIg preparations with higher levels is contraindicated as it can result in severe hypersensitivity and anaphylaxis.

## 4. Discussion

IVIg is an expensive therapy in finite supply as it requires blood donation from healthy donors. Its use in lupus nephritis must be decided upon a case-by-case basis. The treatment of lupus nephritis with IVIg shows promising results in reducing immune deposits in the kidney, reducing proteinuria, improving kidney function, and reducing necessary corticosteroid doses. However, IVIg use has been associated with adverse effects of renal flaring and acute tubular necrosis that can both lead to renal failure. Despite these risks, IVIg treatment appears to be a reasonable option in patients who are refractory to initial induction therapy. Further studies of the use of IVIg as an induction agent for new onset disease as well as renal flares are necessary before its use can be recommended as first line.

The potential mechanisms of action of IVIg are all applicable to patients with lupus nephritis. The pathogenesis of nephritis in lupus includes the accumulation of immune complexes (ICs) between autoantibodies and antigen. IC can form *in situ *or in the circulation. Circulating IC can be passively trapped in the kidney or can be actively bound by renal cells. There is abundant literature demonstrating that IC directly binds to renal parenchymal cells. Heat-aggregated IgG (HA-IgG) localizes to the kidney *in vivo* [77–81] and binds to glomerular mesangial cells [82–91] and glomerular visceral epithelial cells (podocytes) [92] *in vitro* with high specificity. Glomerular IC deposits can be induced in rats and mice by the injection of preformed IC [93–96]. *In vitro*, IC binding to mesangial cells results in IC internalization and activation of specific signaling pathways [83–87], cell proliferation, and release of proinflammatory cytokines and chemokines [90, 91]. Podocytes respond to HA-IgG with altered fibrinolytic activity [97]. Glomerular endothelial cell binding to preformed IC or HA-IgG leads to internalization of autoantibodies [98]. Clearance of pathogenic IC from the kidney by IVIg can act to minimize parenchymal cell activation in lupus patients.

Despite the widely reported findings of IgG accumulation in biopsies of patients with IC kidney diseases, the presence of IC receptors on renal cells has not been firmly established. Binding of IgG by leukocytes is mediated by Fc-receptors (Fc*γ*Rs) [99]. Named for their ability to bind the Fc-region of IgG, they include Fc*γ*RI (activating receptor for monomeric IgG), Fc*γ*RII (inhibitory IC receptor), and Fc*γ*RIII and Fc*γ*RIV (activating IC receptors). Leukocytes internalize IC once bound to Fc*γ*R, resulting in IC degradation or cellular activation. Fc*γ*R activation plays a role in leukocyte migration, proliferation, cytokine production, hypersensitivity reactions, and peripheral tolerance [87]. There is a paucity of human biopsy studies describing parenchymal expression of Fc*γ*Rs [100]. There are numerous reports demonstrating expression of receptors for IgG in cultured mesangial cells [91, 97, 101–107]. Constitutive expression of Fc*γ*RIII has been reported in rat mesangial cells [102]. Stimulating antibodies to rat Fc*γ*RIII activate the same pathways in rat mesangial cells as those activated by preformed IC [102–104]. Fc*γ*RIII has also been identified in human mesangial cells, and activation of this receptor induces cytokine production [103]. Other groups have failed to show basal expression of Fc*γ*RI or Fc*γ*RIII [108], although expression could be measured after stimulation with IFN*γ* and endotoxin [104, 105]. Mouse mesangial cells constitutively express Fc*γ*RII, but Fc*γ*RIII requires stimulation with IFN*γ* [91]. In podocytes, Fc*γ*R does not appear to be expressed, but expression of the neonatal FcR has been reported [109, 110]. All of these receptors could theoretically contribute to the accumulation of IgG in the kidney in SLE. IVIg could potentially alter the balance of inhibitory and activating Fc-receptors in the kidney or could saturate neonatal FcR resulting in more degradation or urinary excretion of pathogenic auto-Abs by the kidney.

There have been several advances in understanding the mechanisms of IVIg. Research into effects of parenchymal FcR may lead to new targets for treating renal manifestations of systemic rheumatologic diseases. Efficacy of recombinant Fc-fragments [111, 112] and sialylated Ig [113] in recapitulating the immunosuppressive effects of IVIg in animal models is revolutionizing the field. The potential development of recombinant reagents may allow for the proper prospective randomized controlled trials that have not been possible with IVIg due to its finite supply. For greatest impact, future studies of IVIg and these newer polyclonal Ig-based derivatives need to include standardized response measures and report specifics on the rates of previously identified adverse events. Patients with lupus nephritis would be an ideal population to perform these studies, as new treatments for lupus nephritis are badly needed. 

## Figures and Tables

**Table 1:** Potential mechanisms of action of IVIg.

(i) Anti-idiotypic binding that neutralizes auto-Abs	
(ii) Competitive inhibition of binding to activating Fc-receptors	
(iii) Upregulation of inhibitory Fc-receptors	
(iv) Delayed clearance of Ab-coated blood cells	
(v) Increased clearance of auto-Abs by reticuloendothelial system	
(vi) Decreased half-life of auto-Abs due to competitive binding to FcRn	

**Table 2:** Studies identified in review of literature on IVIG therapy for lupus nephritis.

Articles retrieved	630
Articles excluded	598
(i) IVIg use not discussed	144
(ii) Use limited to animal/*in vitro *models	49
(iii) Review article	136
(iv) Use in SLE not discussed	38
(v) Use in SLE limited to non-renal disease	217
(vi) Patients reported in subsequent manuscript	5
(vii) Manuscript not available in English*	11
Articles included	32 (22 efficacy/9 adverse effects/1 both)

*Includes 33 patients with lupus nephritis amongst those reported.

**Table 3:** Initial case reports of IVIg therapy of SLE manifestations.

Hjortkjaer Petersen et al. [29]	1990	Pericarditis
Maier et al. [30]	1990	Thrombocyto penia
Tomer and Shoenfeld [31]	1992	Psychosis
Lesprit et al. [32]	1996	Polyneuritis
Aharon et al. [33]	1997	Myelofibrosis
Généreau et al. [34]	1999	Cutaneous lupus
Sherer et al. [35]	1999	Cerebritis
Sherer et al. [36]	1999	Myocarditis
Meissner et al. [37]	2000	Serositis
Sherer et al. [38]	2000	Antiphospholipid syndrome
Hoshi et al. [39]	2004	Pulmonary hemorrhage

**Table 4:** Studies included in IVIg therapy for lupus nephritis.

Study	N*	Study design
Sherer et al. 2008 [28]	62	Cohort, 6 courses LD*
Meissner et al. 2000 [37]	1	Case, 1 course HD
Levy et al. 1999 [46]	5	Cohort, 1–8 courses HD*
Akashi et al. 1990 [48]	2	Cohort, 1–3 courses HD
Oliet et al. 1992 [49]	1	Case, 1 course HD
Winder et al. 1993 [50]	2	Cohort, 10–20 courses HD
Arahata et al. 1999 [51]	1	Case RPGN, 1 course HD
Viertel et al. 2000 [52]	1	Case AKI, 2 courses HD
Gan et al. 2002 [53]	4	Cohort, 1 course HD
Kamali et al. 2005 [54]	4	Cohort, 1–6 courses HD
Micheloud et al. 2006 [55]	1	Case pregnancy, 1 course HD
Levy et al. 2000 [56]	7	Cohort, 1–6 courses HD
Francioni et al. 1994 [57]	12	Cohort, 6–24 courses HD
Monova et al. 2002 [58]	58	Cohort, LD up to 7 years
Bridoux et al. 1998 [59]	3	Cohort AKI/TMA, 1 course HD
Becker et al. 1995 [60]	2	ESRD cohort, courses HD
Lin et al. 1989 [61]	9	Pediatric cohort, 1-2 courses HD
Welch et al. 1995 [62]	1	Case, 6 courses HD (induction)
Silvestris et al. 1996 [63]	3	Cohort, 2 courses HD
Boletis et al. 1999 [64]	14	RCT, 18 courses LD (induction)
Corvetta et al. 1989 [65]	3	Cohort, courses HD
Zandman-Goddard et al. 2012 [47]	11	Cohort, 2–17 courses HD
Chacko et al. 2006 [66]	1	Case, 1 course HD
Tan et al. 2008 [67]	1	Case, 1 course HD
Ng 1999 [68]	1	Case, 1 course HD
Ben-Chetrit et al. 1991 [69]	1	Case, 2 courses HD
Pasatiempo et al. 1994 [70]	3	Cohort, 1 course
Barron et al. 1992 [71]	6	Pediatric cohort
CDC MMWR 1999 [72]	120	Registry, includes non-SLE
Sati et al. 2001 [73]	55	Cohort, includes non-SLE
Orbach et al. 2004 [74]	106	Literature review

*Number of patients in study with lupus nephritis receiving IVIg where efficacy or adverse events could be defined based on information provided. LD: low-dose IVIg, HD: high-dose IVIg.
